# Optimisation and validation of a PCR for antigen receptor rearrangement (PARR) assay to detect clonality in canine lymphoid malignancies

**DOI:** 10.1016/j.vetimm.2016.10.008

**Published:** 2016-12

**Authors:** Elspeth M. Waugh, Alice Gallagher, Hayley Haining, Pamela E.J. Johnston, Francesco Marchesi, Ruth F. Jarrett, Joanna S. Morris

**Affiliations:** a MRC-University of Glasgow Centre for Virus Research, Institute of Infection, Immunity and Inflammation, College of Medical, Veterinary and Life Sciences, 464 Bearsden Road, Glasgow, G61 1QH, UK; b School of Veterinary Medicine, College of Medical, Veterinary and Life Sciences, University of Glasgow, 464 Bearsden Road, Glasgow, G61 1QH, UK

**Keywords:** Canine lymphoma, Genotype, Immunophenotype, Classification, PARR, GeneScan

## Abstract

PCR for antigen receptor gene rearrangements (PARR) analysis is being increasingly used to assist diagnosis of canine lymphoma. In this study, PARR was carried out on consecutive samples received as part of routine diagnostic practice from 271 patients: 195 with lymphoid malignancies, 53 with reactive conditions and 23 with other neoplasms. Initially, published primer sets were used but later minor primer modifications were introduced and primers were rationalised to give a PARR panel that provides a good compromise between sensitivity and cost. Results were compared to diagnoses made by histology or cytology, coupled with immunophenotyping by flow cytometry or immunohistochemistry where possible. After exclusion of 11 poor quality samples, 230/260 (88%) gave a clear result with 162/163 (99%) of samples classified as clonal and 56/67 (84%) classified as polyclonal giving results concordant with the cytological/histological diagnosis. Among 30 samples with equivocal results, 21 had clonal peaks in a polyclonal background and nine showed little amplification. These were from patients with a range of neoplastic and non-neoplastic conditions emphasising the need to interpret such results carefully in concert with other diagnostic tests. The combination of primer sets used in this study resulted in a robust, highly specific and sensitive assay for detecting clonality.

## Highlights

• Ten primer sets detected clonality with high specificity and sensitivity.
• Four extra primer sets may detect clonality in samples with equivocal results.
• Knowledge of sample quality is needed for interpretation of results.
• Samples generating dominant peaks require careful interpretation.

## 1 Introduction

PCR for antigen receptor gene rearrangements (PARR) is increasingly being used in the diagnostic setting as a means of assessing clonality in samples with a differential diagnosis of canine lymphoma. Several different primers sets for amplification of rearranged immunoglobulin heavy chain (IgH) and T-cell receptor gamma (TCRγ) genes have been described (Vernau and Moore, 1999, Burnett et al., 2003, Tamura et al., 2006, Yagihara et al., 2007, Gentilini et al., 2009, Chaubert et al., 2010, Keller and Moore, 2012). Primer design was initially based on cDNA sequences generated by 5′ rapid amplification of cDNA ends from normal canine spleen (Vernau and Moore, 1999, Burnett et al., 2003). High rates of detection of clonality were reported despite the use of a small number of primer sets, suggesting that some gene segments are commonly rearranged or that dogs have a more limited recombinatorial repertoire than humans. Following publication of the canine genome (Lindblad-Toh et al., 2005), primers were modified and added to provide better gene coverage and improve sensitivity (Tamura et al., 2006, Yagihara et al., 2007, Chaubert et al., 2010); however, full annotations of the TCRγ and IgH loci were not published until 2009 and 2010, respectively (Massari et al., 2009, Bao et al., 2010). Studies prior to this did not consider all described segments, with only a single publication using a comprehensive PCR strategy addressing all known gene segments, and this only for TCRγ (Keller and Moore, 2012). It is therefore unlikely that currently used primer sets will detect all antigen receptor gene rearrangements, resulting in sub-optimal sensitivity.

While assay sensitivity generally appears good, with detection of clonality in up to 98% of canine lymphomas (Gentilini et al., 2009), most PARR studies have tested primer sets on selected, well-defined samples from confirmed lymphoid malignancies. In the clinic, however, PARR is most useful in cases where there is some ambiguity over the morphological diagnosis. The performance of these assays with a range of diagnostic samples, including those which are difficult to classify with routine methods, remains to be established. From the limited information available, detection rates in diagnostic samples appear lower. One laboratory reported sensitivity of 75% using the Burnett primers over one year (Avery, 2009), while the comprehensive TCRγ assay yielded concordant results in only 41 of 60 (68%) clinical samples (Keller and Moore, 2012). Thus there is scope to increase the accuracy of these assays through further improvements in primer design. Furthermore, while most studies have assessed sensitivity, specificity of the canine assays has not been widely discussed and most studies have analysed only a few normal or reactive samples, generally in the initial set-up phase. Burnett et al. (2003) detected one clonal IgH rearrangement (an Ehrlichiosis case) in the analysis of 24 samples from dogs with various inflammatory and non-lymphoproliferative neoplastic conditions and Chaubert et al. (2010) observed no clonal TCRγ rearrangements in 23 samples from reactive skin conditions. Although both results suggest that specificity is good, consistent with the 94% reported by Avery (2009), this again requires broader study in clinical samples. While improvements in primer design should enhance sensitivity and specificity, a consolidated interpretation of results obtained from critically reviewing PARR results alongside other diagnostic tests will also impact on the accuracy of these assays.

The aim of this study was to refine and rationalise previously published PARR primer sets in order to produce a panel of assays for routine use in the diagnostic setting. Initially, multiple published PARR primers were used to establish baseline sensitivity and specificity and, subsequently, modifications to both primer sequences and primer combinations were made. Assay conditions were also streamlined to facilitate use in a diagnostic setting. Diagnostic samples from 271 non-selected, suspected lymphoma patients were used in these analyses and results compared with morphological and, where possible, immunophenotypic diagnosis. Estimates of sensitivity and specificity were determined, and common difficulties in interpretation identified.

## 2 Materials and methods

### 2.1 Patient and sample characteristics

Clinical samples were collected from untreated patients presenting with suspected lymphoma to the University of Glasgow Small Animal Hospital, UK. Ethical approval was obtained from the Faculty of Veterinary Medicine Ethics and Welfare Committee, University of Glasgow. Samples consisted of excess material taken as part of routine evaluations at the time of diagnosis and were from 195 patients diagnosed with lymphoid malignancies (172 lymphoma, 11 leukaemia, nine leukaemia/lymphoma, and three plasma cell tumour), 53 with reactive or inflammatory conditions, and 23 with other neoplasms. Samples were either fresh (fine needle aspirates, biopsies, blood or body fluids), or fixed (formalin-fixed, paraffin-embedded (FFPE) biopsies or acetone-fixed, May-Grünwald-Giemsa-stained cytological smears). Most were from lymph node although a range of other tissues was sampled. Patient and sample characteristics are described in Table 1. A wide range of breeds was represented with Crossbreed and Labrador the most common across all groups.

With the exception of 18 samples used in initial assay set-up (5 reactive, 13 lymphoma, all FFPE), all samples were submitted as non-selected diagnostic samples; analysis and interpretation were carried out without knowledge of the diagnosis. Diagnoses were made by cytology or histopathology, with immunophenotyping by flow cytometry (FC) or immunohistochemistry (IHC) where possible. For FC, fresh samples were stained with the antibodies listed in Table 2 and analysed on a Beckman Coulter FC500 flow cytometer using CXP software (Beckman Coulter (UK) Ltd, High Wycombe, UK). Erythrocytes were lysed using CalLyse (Life Technologies Ltd, Paisley, UK). Leukoperm (AbD Serotec, Kidlington, UK) was used prior to staining for internal antigens. Cell populations were gated based on the forward versus side scatter plot and events regarded as positive where the fluorescence intensity was greater than the isotype-matched control for that sample. Where >70% of cells stained with a single antibody or group of antibodies, the cell population was considered to be of that phenotype. IHC on FFPE samples was carried out by Veterinary Diagnostic Services, University of Glasgow. Sections were stained with antibodies (all Dako UK Ltd, Ely, UK) against CD3 (T-cell; polyclonal) and either CD79a (B-cell; clone JCB117) or PAX5 (B-cell; clone DAKPax5) in a Dako Autostainer using the Dako EnVision + ™ System.

Samples diagnosed as null-cell lymphoma (n = 6) were classified as lymphoma on morphological grounds but were negative for CD3 and CD79a/PAX5 by IHC, while one sample diagnosed as ‘double-labelled lymphoma’ was positive for both CD3 and PAX5. CD34-positive leukaemias (n = 6) were CD34 positive by FC and negative for other leukocyte antigens. Cases of lymphoma or leukaemia that were not subjected to immunophenotyping (n = 48) or where immunophenotyping by FC was inconclusive (n = 5) were designated ‘not otherwise specified’ (NOS). Plasma cell tumours (n = 3) were classified on the basis of morphology.

### 2.2 PARR

#### 2.2.1 Primers

Primers were synthesised according to published sequences (Burnett et al., 2003, Tamura et al., 2006, Yagihara et al., 2007, Gentilini et al., 2009, Chaubert et al., 2010), and initially tested in their original pairings (Tables S1 and S2). Each 5′ primer was fluorescently labelled to facilitate visualisation of products by GeneScan analysis. Primers were subsequently compared to recent annotations of the canine IgH (Bao et al., 2010) and TCRγ (Massari et al., 2009) variable region loci. For each primer, the number and location of mismatches to the germline sequence of the IgH locus (accession number NW_003726071) or TCRγ locus (accession number NW_876265), were noted. Mismatches at the 3′ end will have the most detrimental effect on reaction efficiency (Stadhouders et al., 2010), and so we ensured that all retained primers had a perfect match in the five most 3′ bases.

Four IgH V segment primers were retained in our PARR panel: two IgH framework region 1 (FWR1) primers (918 and 919) were included to help mitigate the problem of junctional mutation or somatic hypermutation disrupting the IgH framework region 3 (FWR3) primer binding site, and two IgH FWR3 primers (913 and 916) were retained as they will detect rearrangements in samples with fragmented DNA. Although some germline V segments have a perfect match to only one of these primers, most match more than one of the primers providing some redundancy and mitigating against false negative results. Two IgH J segment primers are required to bind to all rearrangements. A single J1-2 primer (914) was selected from the three tested, and a mismatch in the J3 primer corrected (915a).

Two TCRγ V segment and three J segment primers were included in our final panel. Primer 928 should bind to TCRγ V2 and V6 segments. Following correction of four mismatches at the 5′ end of primer 925 (925a), this primer should bind to V3, V5 or V7 segments. Primer 930, which should bind the majority of TCRγ Jx-2 segments, was modified to include two mixed bases in the middle of the primer to allow for sequence variation between segments (930a). Primer 929 should bind well to Jx-1 segments, except for segment J5-1 where several mismatches occur at the 3′ end; primer 931 was therefore designed to match J5-1 more closely. The final PARR primer sequences are listed in Table 3, Table 4.

The amplifiability of all DNA samples was assessed using two control PCRs. Initially Cμ (128 bp) and γ-actin (272 bp) primers were used, and later γ-actin (125 and 450 bp) primers; amplicon sizes correspond to those generated by the PARR primer sets (Table 4).

#### 2.2.2 PCR and analysis

DNA was extracted using QIAamp DNA Mini kits or DNA FFPE Tissue kits (Qiagen Ltd, Manchester, UK). Reactions were performed in a total volume of 25 μl, and contained 100 ng DNA (where possible), each primer at 250 nM (IDT, Leuven, Belgium) and 1 x HotStarTaq Plus Master Mix (Qiagen). Thermal cycling was carried out on a GeneAmp PCR System 9700 (Applied Biosystems, Life Technologies). Initially, all primer sets used identical thermal cycling conditions: 95 °C for 5 min, followed by 40 cycles of 95 °C for 30 s, 58 °C for 30 s, 72 °C for 30 s, with a final extension of 72 °C for 30 min to facilitate addition of terminal adenosine bases on each PCR product. Following optimisation, the annealing temperature for primer sets 9, 10, 13 and 14 only was reduced to 50 °C consistent with the low melting temperatures of primers 929 and 931.

Fragment analysis was carried out using GeneScan methodology on an ABI 3130×l Genetic Analyser (Applied Biosystems) with a 36 cm capillary length loaded with POP-4 polymer. Products amplified with primer sets 1 and 4, 2 and 5, and 3 and 6, were combined prior to fragment analysis; 5′ primers in each combination were labelled with different fluorochromes so that reaction products could be distinguished (Table 3).

### 2.3 Scoring and interpretation

**Fig. 1:**
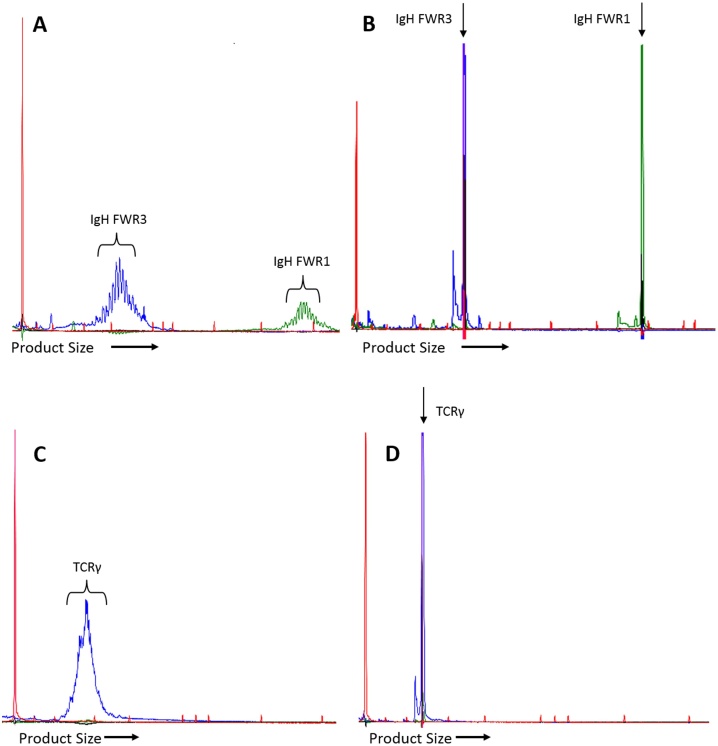
Typical PARR electropherograms showing polyclonal and clonal products. (For interpretation of the references to colour in this figure legend, the reader is referred to the web version of this article.) Size markers are shown in red. A: Polyclonal products from two separate IgH reactions from the same sample. Fragments are multiplexed for GeneScan analysis with the shorter FWR3 fragments (blue; primer set 1) on the left and the longer FWR1 fragments (green; primer set 4) on the right. There is a Gaussian distribution of products with a peak every three bases. B: Clonal products from a B-cell lymphoma using the same primer sets as in A. A single tall sharp peak is seen. Occasionally a smaller secondary peak is seen at the base to the left; this feature is due to overloading of the capillary with product and disappears if the product is diluted. C: Polyclonal TCRγ products (blue; primer set 7) exhibiting a Gaussian distribution without a peak every 3 bases. A slightly irregular jagged appearance can sometimes occur. D: Clonal TCRγ product (primer set 7) with a similar appearance to the IgH peaks in B.

**Fig. 2:**
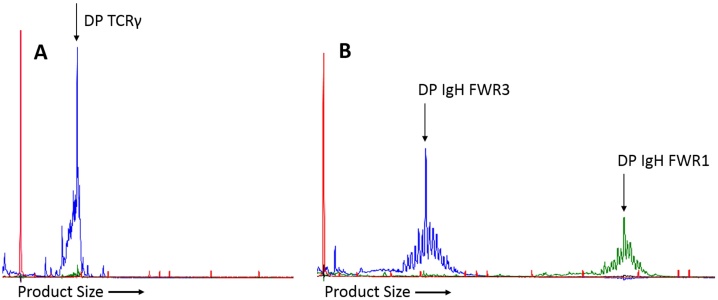
Amplification of dominant peaks. (For interpretation of the references to colour in this figure legend, the reader is referred to the web version of this article.) Size markers are shown in red. A: Dominant peak with TCRγ primer set 9 (blue) from a T-cell lymphoma. The background polyclonal distribution is skewed towards the peak. Testing with additional TCRγ primer sets in the final PARR protocol revealed a clonal T-cell population (primer set 10). B: Dominant peaks with IgH primer sets 1 (blue) and 4 (green) from a SMZL. A clear dominant peak is seen with both sets, arising from the same area of the polyclonal distribution, suggesting they represent the same clonal subpopulation.

**Fig. 3:**
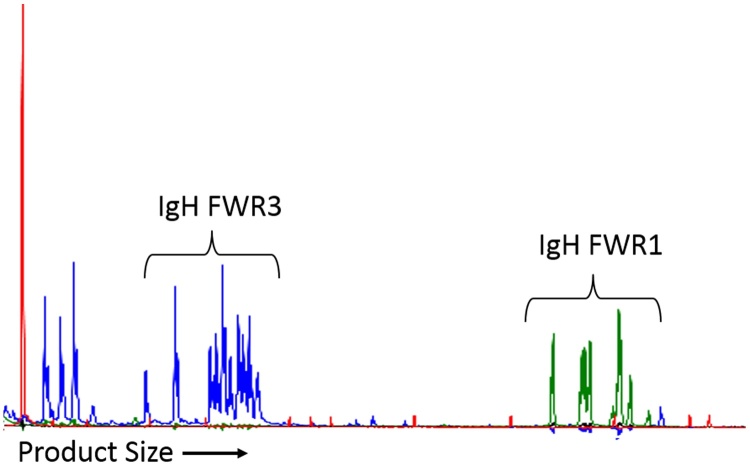
PARR electropherogram showing poor amplification. (For interpretation of the references to colour in this figure legend, the reader is referred to the web version of this article.) Size markers are shown in red. This sample has amplified poorly, generating a small number of low amplitude peaks using both primer sets 1 (blue) and 4 (green). The lack of a Gaussian distribution of fragments or a clonal peak suggests that only a small number of cells in the sample contained rearranged IgH genes. This sample was classified in the ‘no peak’ category, as the result suggests that either few lymphoid cells were present in the sample or that a clonal rearrangement was present but was not detected using these primers.

For each individual PARR primer set, results were subjectively scored as clonal (one or two tall clear peaks with minimal polyclonal amplification around the base of the peak (Fig. 1B, 1D)); polyclonal (fragments distributed over the size range of the primer set, either in a Gaussian distribution or a slightly more skewed distribution (Fig. 1A, C)); dominant peak (one or more clear peaks visible within a polyclonal distribution (Fig. 2)); poor amplification (several small peaks or an irregular polyclonal distribution of low amplitude (Fig. 3)); or negative (no amplification products in the appropriate size range).

Based on the overall pattern of results with all the PARR primer sets and the amplifiability controls, samples were then assigned to one of seven categories (Table 5): ‘B-cell clone’; ‘T-cell clone’; ‘clonal IgH and TCR rearrangement’; ‘reactive’; ‘dominant peak only’; ‘no peak’; and ‘inadequate sample’. Samples were classed as clonal if a clear clonal amplification product was seen, even if the larger of the two DNA amplifiability controls did not work. If no clear clonal or polyclonal product was detected in such cases, the sample was categorised as ‘inadequate sample’ since amplification of the FWR1 IgH primer sets could not be assessed and therefore results could not be interpreted with confidence. Samples categorised as ‘inadequate sample’ were excluded from sensitivity and specificity analyses. Samples were assigned to the ‘dominant peak only’ category if there was a clonal peak superimposed on a polyclonal background with no evidence of a discrete clonal peak using any of the primer sets. Samples were classified as ‘no peak’ when the control PCRs had worked satisfactorily but the individual PCRs either generated no amplification products or showed poor amplification; this pattern is observed in samples which contain few lymphocytes and also in samples with clonal rearrangements that are not amplified by the PARR primers.

## 3 Results

### 3.1 Initial results

Using the published primer sets in their original pairings (Tables S1 and S2), the majority of samples diagnosed as B-cell lymphoma had detectable clonal IgH rearrangements (73 of 81; 90.1%; see Table 6). A smaller proportion of the T-cell lymphomas had identifiable clonal TCRγ rearrangements (29 of 39; 74.4%), and only 72.9% (35 of 48) of lymphoma NOS samples had a detectable clone. Since these findings suggested that some rearrangements, particularly of TCRγ, were being missed a systematic review of the primer sets was undertaken to try and improve sensitivity, as described in Materials and Methods. After minor modifications to the primer sequences and rationalisation of use of the highly similar J1-2 primers, 14 primer sets were selected; ten primer sets were included in our standard panel (Table 3) and four sets formed an additional panel (Table 4).

### 3.2 Final results

Following primer review, samples with no clear clonal result were further analysed using the new primer set combinations (IgH set 6, TCRγ sets 8 and 10) and with TCRγ primer set 9 using the new thermal cycling conditions. Samples previously designated as ‘reactive’, ‘inadequate sample’, ‘no peak’, or ‘dominant peak only’ were assayed. Since further testing was not performed on samples that had a clear clonal result, each sample was not tested with every primer set. All samples submitted following primer review were tested with the standard panel of ten primer sets (six IgH and four TCRγ, Table 3). If a clear clonal result was still not detected, samples were further tested using the four additional primer sets (two IgH and two TCRγ, Table 4). All 271 samples were assigned to a final results category (Table 6).

Eleven out of the two hundred and seventy one samples (Table 6) were designated ‘inadequate sample’ and excluded from further analysis based on poor or no amplification with at least one amplification control and the PARR primers. After primer modification, one additional ‘inadequate sample’ was reclassified, since primer set 9 generated a clonal TCRγ product using the new cycling conditions (negative using previous conditions). Amplification of a specific TCRγ product was confirmed by sequencing (data not shown). Of the remaining 260 samples, 163 (62.7%) had a clear clonal result and 67 (25.8%) were clearly polyclonal. Nine samples (3.5%) had poor or negative amplification with all PARR primer sets despite good amplification with the control primers (Fig. 3), suggesting that the samples contained few lymphocytes or a clonal rearrangement had been missed, while 21 samples (8.1%) had one or more dominant peaks (Fig. 2) with no clear evidence of clonality.

**Fig. 4:**
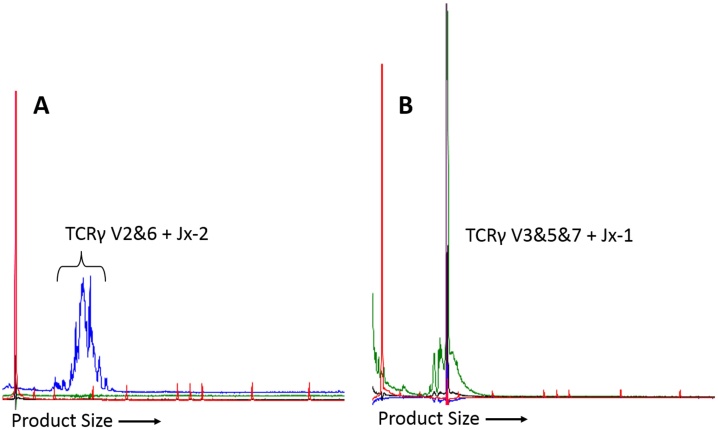
PARR electropherograms of a T-cell lymphoma. (For interpretation of the references to colour in this figure legend, the reader is referred to the web version of this article.) Size markers are shown in red. A: Original primer sets. This sample was designated as reactive due to polyclonal amplification using the TCRγ primer sets. Primer set 7 is shown (blue). B: Final primer sets. Clonal product detected using one of the TCRγ primer sets added in the final protocol (primer set 10; green). V2&6, Jx-2, V3&5&7 and Jx-1 refer to the TCRγ V and J segments amplified by the primer set (see Table 3).

Introduction of the modified primer sets led to re-categorisation of 17 (6.3%) samples. Fifteen of these samples had detectable clonal TCRγ rearrangements (Fig. 4), and were previously classified as ‘reactive’ (n = 8), ‘dominant peak only’ (n = 4), ‘no peak’ (n = 2) or ‘inadequate sample’ (n = 1, as described above). All TCRγ clones were detected using primer sets in the standard panel, however in two samples a clearer clonal peak was generated using additional primer sets 13 or 14. The remaining two re-categorised samples had clonal IgH rearrangements detected using additional primer set 11; these cases had previously been classified as ‘reactive’ and ‘dominant peak only’.

### 3.3 Comparison with morphological diagnosis

Final results were then compared with the diagnosis reached on the basis of morphology. Results were clearly discordant in 12 samples (4.6%). There was one false positive result; a patient diagnosed with myeloid neoplasia had clonal peaks detected using two IgH primer sets. The clonal result was repeatable using the same sample (lymph node); however, fresh samples from both lymph node and bone marrow yielded polyclonal results. Concurrent cytology indicated that the lymph node contained a high proportion of myeloid precursors amidst a background of mature lymphoid cells. Eleven samples were diagnosed as lymphoid malignancy but had a polyclonal PARR result and could represent false negative results. These comprised two splenic B-cell lymphomas, four lymphoma NOS samples, two null-cell lymphomas, and three CD34-positive leukaemias. All four ‘diagnoses’ were over-represented in this group compared with the overall sample distribution. A further 12 samples diagnosed as lymphoid malignancy had equivocal PARR results: eight were ‘dominant peak only’ and four were categorised as ‘no peak’.

Overall, 160 of 185 samples with a diagnosis of lymphoid malignancy produced a clonal result (B-cell, T-cell or both) with the standard panel of ten primer sets, or 162 of 185 samples when the additional primer sets were included. This gives sensitivities of 86.5% and 87.6%, respectively. Specificity was 98.7%, based on the absence of clonal IgH or TCR rearrangement in 74 of 75 samples with conditions other than lymphoid malignancy.

### 3.4 Concordance between immunophenotype and genotype

Conclusive immunophenotyping results were available for 139 of 195 lymphoid malignancy cases (Table 1). Of these, 126 had a defined B- or T-cell immunophenotype, which allowed a direct comparison with the PARR genotype. A clonal PARR result was generated in 112 (88.9%) of these samples; the remaining 14 samples yielded either polyclonal or equivocal results (Table 6). In 109 of the 112 clonal samples (97%), PARR genotype was concordant with the recorded immunophenotype. In three samples there was potential evidence of cross-lineage rearrangement. One sample diagnosed as T-cell lymphoma by IHC had a clonal rearrangement detected using three IgH primer sets, suggesting a B-cell origin. All TCR primer sets yielded polyclonal products. The diagnosis of non-epitheliotropic cutaneous T-cell lymphoma was based on CD3 positivity, but approximately 50% of the infiltrating cells did not stain with CD3 or PAX5 antibodies and could have represented a neoplastic population of B-cells with clonal IgH rearrangement and down-regulation of PAX5. Material was not available for further IHC analysis with CD79a antibody, or repeat DNA extraction and PARR analysis. Two samples diagnosed as B-cell lymphoma by IHC had clonal results using both IgH and TCRγ primer sets, and so a definitive lineage could not be assigned using PARR. While in both samples a small polyclonal background was present, the clonal peaks generated with the TCRγ primer sets were of sufficient amplitude that both samples could have been diagnosed as T-cell lymphoma had no concurrent IgH peaks been present and had the IHC not been performed.

### 3.5 Lineage classification determined by PARR

Six samples were classified as B- or T-cell lineage using PARR where there was no clearly defined B- or T-cell population following immunophenotyping. Of these, three were diagnosed as null-cell (non-B, non-T) lymphoma by IHC and PARR identified one as a B-cell and two as T-cell tumours. Similarly three samples classified as CD34-positive leukaemia with dim or negative staining for leukocyte antigens on FC were assigned to a T-cell lineage by PARR. Lineage was also assigned to the five samples previously designated as lymphoma NOS where FC was inconclusive.

## 4 Discussion

The aim of this study was to develop a panel of PARR assays for routine use in the diagnostic setting. As a starting point we used PCR assays previously described in the literature but later modified some of the primer sequences, rationalised the use of highly similar primers, and adapted the thermal cycling parameters to allow use of a minimum number of different conditions. These changes improved the ability to detect clonal rearrangements, particularly in T-cell malignancies. We describe a ‘standard’ panel of ten primer pairs, recommended for routine use. These comprise six IgH and four TCR primer sets, which will pick up the vast majority of rearrangements. A further four additional primer pairs are described that are useful in certain circumstances.

Comparison with morphological diagnoses suggested that the sensitivity of this PARR panel was 86.5%, which is within the range (80–97.9%) described elsewhere (Burnett et al., 2003, Gentilini et al., 2009, Chaubert et al., 2010, Keller and Moore, 2012). However, previous studies have generally used samples from known cases of lymphoma, excluding samples without a confirmed diagnosis and sometimes also lymphoid leukaemias and plasma cell tumours (Burnett et al., 2003, Gentilini et al., 2009). In particular, samples from other neoplastic conditions have often been avoided, despite the fact that lymphoma may be a differential diagnosis in many of these cases. By contrast, this case series was composed of unselected diagnostic samples from a wide range of source materials, to assess the usefulness of PARR in routine diagnostic practice.

Assay sensitivity improved as more primer sets were included, offset by increased cost of labour and reagents. We believe that the standard protocol described here is a good compromise between sensitivity and ease of use and has improved sensitivity over previous studies using fewer primer sets (Burnett et al., 2003, Tamura et al., 2006, Chaubert et al., 2010). Retaining singleplex reactions avoids difficulties in interpretation and should allow detection of smaller clonal populations; using multiple primer sets also provides redundancy should mutation alter any primer binding site. In part, the number of primer sets in the standard panel was chosen for practicality, as with multiplexing at the analysis stage this allowed the use of a single column of a 96-well plate per sample in the GeneScan analysis. The additional primer sets increased overall sensitivity slightly, but not enough to justify the added expense of running them routinely. Instead, these primer combinations were reserved for samples where no clone was detected but there was a high index of suspicion for lymphoma. Samples with a dominant peak may also warrant the use of the additional sets, particularly where the dominant peak is detected using primer 929 (primer sets 9 and 10) as this shares similarities with primer 931 (additional primer sets 13 and 14).

There are several plausible explanations for our failure to detect clonal rearrangements in samples with a morphological diagnosis of lymphoma. One obvious concern is that the primers used do not bind to the rearranged gene segments (Valli et al., 2006, O'Brien et al., 2013). Analysis of the described functional V and J segments of IgH and TCRγ indicates that there are few gaps in coverage. Failure of primer binding is therefore more likely to be due to somatic hypermutation. Certain diagnoses were clearly over-represented in this group of samples, including splenic B-cell lymphoma, null cell lymphoma and CD34-positive leukaemia. Sampling error leading to masking of a focal neoplastic lesion by a polyclonal signal in an organ rich in normal lymphocytes may explain the over-representation of splenic B-cell lymphomas (Burnett et al., 2003). In null cell lymphomas and CD34-positive leukaemias there may be a genuine absence of IgH or TCRγ loci rearrangements since null-cell lymphomas may actually be NK cell tumours (van Dongen et al., 2003, Ponce et al., 2010), and CD34-positive leukaemias may represent expansions of immature B-cells that have not completed V(D)J recombination. This would appear as ‘no peak’ when most cells are non-B-, non-T-cells, or as a polyclonal result when residual reactive B- and T-cells are present. In all these cases, consideration should also be given to the accuracy of the original diagnosis. Although the cytological or histological diagnosis was regarded as definitive for the purposes of this analysis, arriving at the most accurate diagnosis requires consideration of all available diagnostic tests.

False positive results were rare (1 of 75), giving a specificity of 98.7%. Clonal IgH peaks were detected in a sample from a myeloid neoplasia. Although rearrangement of IgH has been documented in human and canine acute myeloid leukaemia (Cheng et al., 1986, Kyoda et al., 1997), it is possible that the clonal products in this sample resulted from sampling error, since repeated testing of fresh samples gave polyclonal results (Elenitoba-Johnson et al., 2000, Bienzle and Vernau, 2011, Langerak et al., 2012). For cost reasons, we do not routinely perform testing of samples in duplicate as has been recommended (Keller et al., 2016) but where pseudoclonality is suspected or PARR gives unexpected results repeat testing of the same or additional samples should be performed.

Despite the use of routine diagnostic samples, only 11 of 271 (4%) samples were classified as inadequate. With one exception, these samples were extracted from FFPE material, which is likely to have contained more degraded DNA than the fresh tissue (van Dongen et al., 2003), or may have contained PCR inhibitors (An and Fleming, 1991). Nevertheless, fixed material generally performed well, with 79 FFPE and nine fixed smear samples producing amplifiable DNA.

Five of the nine samples placed in the ‘no peak’ category were from patients with non-lymphoid neoplasia, consistent with the PARR result. Two samples with clonal T-cell peaks were identified using only the new, modified primer sets and had previously been categorised as ‘no peak’. Results must be interpreted alongside findings using other diagnostic modalities and if this pattern is seen in samples where there is a high index of suspicion of lymphoma, repeat sampling and use of additional PARR primer sets are recommended.

Perhaps the most problematic samples to interpret were those in the ‘dominant peak only’ category, with direct impact on overall assay sensitivity. Most previous reports either do not mention this phenomenon, or infer that all such results indicate clonality (Burnett et al., 2003, Lana et al., 2006, Gentilini et al., 2009). Keller and Moore (2012) defined any dominant peak with a peak height at least twice as tall as the polyclonal background as significant. Using this definition all ‘dominant peak only’ samples in this series would have been classified as clonal, increasing sensitivity to 97.6% but with a concomitant drop in specificity to 84.3%. These results lead us to concur with the recommendation made in a recent review of PARR (Keller et al., 2016), which concludes that mathematical algorithms such as this cannot be used to aid interpretation of PARR results.

Dominant peaks were often observed in samples with a clear clonal peak detected with another primer set, suggesting that primer mismatches, most probably resulting from somatic hypermutation, can give rise to this pattern on electropherograms. This again emphasises the benefit of using multiple primer sets, including the additional primer sets when there is a high index of suspicion. A dominant peak may also indicate a small clonal population of cells within a background of reactive lymphocytes (Gentilini et al., 2009, Keller and Moore, 2012) either in early neoplasia, or as feature of particular tumours where neoplastic cells do not replace the whole organ, such as splenic marginal zone lymphoma (SMZL). Here, this could be seen in two samples of SMZL and one of low-grade lymphoma which generated dominant peaks using at least one IgH primer set.

Dominant peaks could also result from antigenic stimulation occurring during infection or inflammatory reactions, as seen in seven reactive/inflammatory samples in this series. Non-neoplastic clonal proliferations have been documented in humans (Posnett et al., 1994, Billadeau et al., 1996) and in dogs, where infection with *Ehrlichia* species may generate clonal PARR results (Burnett et al., 2003). One reactive sample classed as ‘dominant peak only’ was a dog with suspected tick-borne disease; unfortunately there was no opportunity to test a second sample from this dog post-treatment. Follow-up and repeat sampling of patients with samples displaying dominant peaks may be required to help establish their significance, particularly in cases where an inflammatory lesion could progress to overt lymphoma, such as inflammatory bowel disease. In one T-cell lymphoma, a dominant peak of the opposite genotype (IgH) was the only evidence of clonality while for two other lymphomas with similar dominant peak results, a clone of the ‘correct’ genotype was detected only after using the additional primer sets. Cross-lineage dominant peaks were also seen in 17 samples with a clonal result (11 B-cell and six T-cell). While sampling error leading to pseudoclonality could account for some of these results, they may also be due to a restricted antigenic response to the neoplastic cells.

The agreement between PARR and previous immunophenotype was excellent (97%). Two of three discordant samples had clonal rearrangements of both IgH and TCRγ, which has been documented previously in canine and human lymphoid tumours (Burnett et al., 2003, Tan et al., 2006, Valli et al., 2006, Bagg, 2006). In humans, the clonal rearrangements may arise from separate populations of cells. In T-cell tumours, a clonal B-cell population may arise secondary to immune dysfunction, usually in association with EBV infection (Luzzatto et al., 2005, Tan et al., 2006) and transform to produce a tumour containing malignant B- and T-cells (Zettl et al., 2002). In B-cell tumours, a restricted T-cell response may generate clonal TCR rearrangements (Sze, 2005). Alternatively, IgH and TCR rearrangements may occur in the same early precursor cell (Bagg, 2006).

In this case series, PARR proved useful for assigning lineage where other methods were inconclusive. A previous study reported that FC more accurately determines lineage (Thalheim et al., 2013); however, fewer PARR primer sets were used in the latter study, potentially limiting assay sensitivity. Where surface antigens are down-regulated, or the malignant cell population is not the most numerous in the sample (for example T-cell-rich B-cell lymphoma), PARR will define lineage more accurately than FC. While previous studies have suggested that PARR should not be used as a means of assigning cell lineage because of problems with cross-lineage rearrangement, our results indicate that clonal cross-lineage rearrangement was rare in this case series. We would suggest that where other modalities for immunophenotyping are not available, PARR is an appropriate tool for lineage determination.

## 5 Conclusions

The combination of primer sets used in this protocol resulted in a robust, highly sensitive and specific assay. Although PARR provides diagnostic information unavailable from other tests and can help determine tumour lineage where other techniques have failed, interpretation of results must consider clinical presentation, cell morphology, immunophenotype and other ancillary tests. Knowledge of sample quality is essential, as samples with few cells or poor quality DNA will likely amplify poorly, giving an equivocal result. Dominant peaks, which may indicate a neoplastic population within a reactive background, but are also seen in non-lymphoma samples, pose a particular challenge. Better understanding of the nature of these peaks may come from longer term follow-up of cases and sequencing of products. Further investigation of these findings will improve the precision of this important diagnostic test.

## Figures and Tables

**Table 1:** Patient and sample characteristics.

Diagnosis	Number	Sample type	Site	Sex^a^ (F:FN:M:MN)	Age^a^ (median)	Age^a^ (range)
B-cell lymphoma/leukaemia	81	Fresh	LN (46); B (3); Sp (1); AF (1)	7:19:25:22	7y 10m	1y 3 m–14y
		FFPE	LN (25); Sp (5)			
T-cell lymphoma/leukaemia	45	Fresh	LN (17); B (5); BM (4)	6:15:9:14	8y	2y 5 m–12y 7m
		FFPE	LN (10); Skin (7); GIT (1); MM (1)			
Lymphoma/leukaemia NOS	53	Fresh	LN (20); B (3); MM (3); CSF (2); GIT (1), L (1); AF (1); AH (1), PE (1)	7:17:9:17	6y 10m	3y–16y 8m
		FFPE/fixed	LN (10); GIT (6); Sp (2); Skin (2)			
Null-cell lymphoma	6	Fresh	LN (1)	1:2:2:0	9y	4y–12y
		FFPE	LN (3); Skin (1); L (1)			
Double-labelled lymphoma	1	FFPE	LN (1)	1:0:0:0	10y	10y
Plasma cell tumour	3	Fresh	LN (2); BM (1)	1:1:0:1	11y 11m	6y 2 m–13y
CD34-positive leukaemia	6	Fresh	B (5); BM (1)	2:1:1:1	10y	3y–10y 8m
Reactive/inflammatory condition	53	Fresh	LN (17); CSF (7); B (4); Sp (2); BM (2); GIT (2); Mass (1), AH (1)	9:8:19:15	5y 11m	9 m–14y
		FFPE/fixed	LN (14); GIT (2); Sp (1)			
Other neoplasms^b^	23	Fresh	LN (3); B (3); Mass (3); Sp (2); MM (2); BM (1); Iris (1); CSF (1); BAL (1)	5:5:6:6	6y 4m	4 m–14y
		FFPE/fixed	Skin (3); LN (1); Mass (1); Brain (1)			

NOS: not otherwise specified; LN: lymph node; B: blood; Sp: spleen; AF: abdominal fluid; BM: bone marrow; GIT: gastrointestinal tract; MM: mediastinal mass; CSF: cerebrospinal fluid; L: liver; AH: aqueous humour; PE: pericardial effusion; BAL: bronchoalveolar lavage; F: female; FN: female neutered; M: male; MN: male neutered; y: years; m: months.

a Sex and age were not known for all dogs.

b Other neoplasms comprised histiocytic neoplasms (7), carcinoma (4), myeloid neoplasms (3), thymoma (3), sarcoma (2), osteosarcoma (1), thyroid neoplasm (1) and undefined (2).

**Table 2:** Antibodies used for flow cytometric analysis of canine samples .

Antibody	Cellular Target	Clone
Isotype-matched triple negative FITC/PE/Alexa647	Negative control	
CD5-FITC	Pan T-cell	YKIX322.3
CD21-PE	Mature B-cell	CA2.1D6
CD45-Alexa647	Pan leukocyte	YKIX716.13
CD3-FITC	Pan T-cell	CA17.2A12
CD4-PE	Helper T-cell	YKIX302.9
CD8-Alexa647	Cytotoxic T-cell	YCATE55.9
CD34-FITC	Stem/precursor cells	2E9
CD79a-Alexa647^a^	Pan B-cell	HM57
MHC II-FITC	Activated lymphocytes	YKIX334.2
MAC387-FITC^a^	Macrophages and monocytes	MAC387
CD14-Alexa647	Monocytes	TUK4

FC antibodies were sourced from AbD Serotec, with the exception of CD34-FITC (Santa Cruz Biotechnology Inc, Heidelberg, Germany).

a Antibody targeting an internal epitope (requires permeabilisation).

**Table 3:** PARR primer sets used in the final protocol.

Primer set	Primer number	Target	Original primer name	Reference	Sequence	Product size
1	913	IgH V FWR3	CB1 (5′)	Burnett (2003)	6-FAM-CAG CCT GAG AGC CGA GGA CAC	85–139 bp
	914	IgH J1-2	CB2 (3′)	Burnett (2003)	TGA GGA GAC GGT GAC CAG GGT	
2	916	IgH V FWR3	Tamura F (5′)	Tamura (2006)	6-FAM-ACA CGG CCV TGT ATT ACT GT	79–90 bp
	914	IgH J1-2	CB2 (3′)	Burnett (2003)	TGA GGA GAC GGT GAC CAG GGT	
3	913	IgH V FWR3	CB1 (5′)	Burnett (2003)	6-FAM-CAG CCT GAG AGC CGA GGA CAC	85–145 bp
	915a	IgH J3	CB3(3′)^a^	Burnett (2003)	TGA GGA CAC **G**AA GAG TGA GG	
4	919	IgH V FWR1	3′ FWR1 (5′)	Gentilini (2009)	HEX-GCC TCT GGA TTC ACC TTC AG	262–315 bp
	914	IgH J1-2	CB2 (3′)	Burnett (2003)	TGA GGA GAC GGT GAC CAG GGT	
5	918	IgH V FWR1	5′ FWR1(5′)	Gentilini (2009)	HEX-GAG GTG CAG CTG GTG GAG TCT	309–378 bp
	914	IgH J1-2	CB2 (3′)	Burnett (2003)	TGA GGA GAC GGT GAC CAG GGT	
6	919	IgH V FWR1	3′ FWR1 (5′)	Gentilini (2009)	HEX-GCC TCT GGA TTC ACC TTC AG	262–315 bp
	915a	IgH J3	CB3(3′)^a^	Burnett (2003)	TGA GGA CAC **G**AA GAG TGA GG	
7	928	TCRγ V2&6	dTCRγ-Va (5′)	Chaubert (2010)	6-FAM-GGC GTG TAC TAC TGC GCT GCC	55–82 bp
	930a	TCRγ Jx-2	dTCRγ-Jb (3′)^a^	Chaubert (2010)	TGT GCC AGG ACC AA**D Y**AC TTT	
8	925a	TCRγ V3&5&7	Vγb (5′)^a^	Yagihara (2007)	HEX-**CCA** TGT A**Y**T ACT GTG CCT GCT GG	55–82 bp
	930a	TCRγ Jx-2	dTCRγ-Jb (3′)^a^	Chaubert (2010)	TGT GCC AGG ACC AA**D Y**AC TTT	
9	928	TCRγ V2&6	dTCRγ-Va (5′)	Chaubert (2010)	6-FAM-GGC GTG TAC TAC TGC GCT GCC	55–82 bp
	929	TCRγ Jx-1	dTCRγ-Ja (3′)	Chaubert (2010)	TAC CTT CTG TAA ATA TCT TGA TC	
10	925a	TCRγ V3&5&7	Vγb (5′)^a^	Yagihara (2007)	HEX-**CCA** TGT A**Y**T ACT GTG CCT GCT GG	55–82 bp
	929	TCRγ Jx-1	dTCRγ-Ja (3′)	Chaubert (2010)	TAC CTT CTG TAA ATA TCT TGA TC	

Altered bases are marked in bold. Degenerate bases: V = A, C or G; D = A, G or T; Y = C or T.

a Denotes primer altered slightly from the original published sequence; this is also marked by the suffix ‘a’ in the primer number.

**Table 4:** Additional PARR primer sets and control assays.

Primer set	Primer number	Target	Primer name	Reference	Sequence	Product size
11	916	IgH V FWR3	Tamura F (5′)	Tamura (2006)	6-FAM-ACA CGG CCV TGT ATT ACT GT	79–90 bp
	915a	IgH J3	CB3(3′)^a^	Burnett (2003)	TGA GGA CAC **G**AA GAG TGA GG	
12	918	IgH V FWR1	5′ FWR1(5′)	Gentilini (2009)	HEX-GAG GTG CAG CTG GTG GAG TCT	309–378 bp
	915a	IgH J3	CB3(3′)^a^	Burnett (2003)	TGA GGA CAC **G**AA GAG TGA GG	
13	928	TCRγ V2&6	dTCRγ-Va (5′)	Chaubert (2010)	6-FAM-GGC GTG TAC TAC TGC GCT GCC	55–82 bp
	931	TCRγ J5-1	J5-1		TTC CTT CTG CAA ATA ATC TTG AT	
14	925a	TCRγ V3&5&7	Vγb (5′)^a^	Yagihara (2007)	HEX-**CCA** TGT A**Y**T ACT GTG CCT GCT GG	55–82 bp
	931	TCRγ J5-1	J5-1		TTC CTT CTG CAA ATA ATC TTG AT	
Cμ		IgM constant region	Cμ 1 (5′)	Burnett (2003)	HEX-TTC CCC CTC ATC ACC TGT GA	128 bp
			Cμ 2 (3′)	Burnett (2003)	GGT TGT TGA TTG CAC TGA GG	
γ-actin		Canine γ-actin	γ-actin (5′)	Gentilini (2009)	6-FAM-ACC ACT GGT ATT GTC ATG GAC TCT G	
			γ-actin (3′)		AGG TAG TCA GTC AGG TCC CGG	125 bp
			γ-actin (3′)	Gentilini (2009)	GCT CTT CTC CAG GGA GGA CGA	272 bp
			γ-actin (3′)		CTC TCC CTT AGA GGG CAC ACG	450 bp

Altered bases are marked in bold. Degenerate bases: V = A, C or G; Y = C or T.

a Denotes primer altered slightly from the original published sequence; this is also marked by the suffix ‘a’ in the primer number.

**Table 5:** Categories used to describe samples in PARR interpretation.

Results Category	Definition
B-cell	Clonal amplification with one or more IgH primer sets.
T-cell	Clonal amplification with one or more TCRγ primer sets.
IgH and TCRγ rearrangement	Clonal amplification of similar amplitude with both IgH and TCRγ primer sets.
Reactive	Polyclonal amplification with all PARR primer sets.
Dominant peak only	Dominant peak seen with one or more PARR primer sets with no clear clonal peak.
No peak	Poor or negative amplification with PARR primer sets alongside good amplification of both DNA amplifiability controls. The presence of few lymphocytes in the sample cannot be distinguished from failure to amplify a clonal rearrangement.
Inadequate sample	Poor or negative amplification with one or both DNA amplifiability controls alongside poor or negative amplification with PARR primer sets.

**Table 6:** PARR results by diagnosis.

Diagnosis	PARR Result							
	B-cell	T-cell	Both	Reactive	DP only	No peak	Inadequate sample	Total
B-cell lymphoma/leukaemia	73^a^	0	2	2	4	0	0	81
T-cell lymphoma/leukaemia	1	38	0	0	1	0	5	45
Lymphoma/leukaemia NOS	20	20	0	4	3	1	5	53
Null-cell lymphoma	1	2	0	2	0	1	0	6
Double-labelled lymphoma	0	0	0	0	0	1	0	1
Plasma cell tumour	2	0	0	0	0	1	0	3
CD34-positive leukaemia	0	3	0	3	0	0	0	6
Reactive/inflammatory	0	0	0	46	7	0	0	53
Other neoplasia	1	0	0	10	6	5	1	23
Total	98	63	2	67	21	9	11	271

Both: clonal IgH and TCRγ rearrangement; DP: dominant peak. See Table 5 for definition of PARR results categories.

a Includes two samples where a clone was detected using only the extra primer set 11 (916/915a), not part of the routine PARR protocol.

## References

[bib0005] An S.F., Fleming K.A. (1991). Removal of inhibitor(s) of the polymerase chain reaction from formalin fixed: paraffin wax embedded tissues. J. Clin. Pathol..

[bib0010] Avery A. (2009). Molecular diagnostics of hematologic malignancies. Top. Companion Anim. Med..

[bib0015] Bagg A. (2006). Immunoglobulin and T-cell receptor gene rearrangements: minding your B's and T's in assessing lineage and clonality in neoplastic lymphoproliferative disorders. J. Mol. Diagn..

[bib0020] Bao Y., Guo Y., Xiao S., Zhao Z. (2010). Molecular characterization of the VH repertoire in Canis familiaris. Vet. Immunol. Immunopathol..

[bib0025] Bienzle D., Vernau W. (2011). The diagnostic assessment of canine lymphoma: implications for treatment. Clin. Lab. Med..

[bib0030] Billadeau D., van Ness B., Kimlinger T., Kyle R.A., Therneau T.M., Greipp P.R., Witzig T.E. (1996). Clonal circulating cells are common in plasma cell proliferative disorders: a comparison of monoclonal gammopathy of undetermined significance smoldering multiple myeloma, and active myeloma. Blood.

[bib0035] Burnett R.C., Vernau W., Modiano J.F., Olver C.S., Moore P.F., Avery A.C. (2003). Diagnosis of canine lymphoid neoplasia using clonal rearrangements of antigen receptor genes. Vet. Pathol..

[bib0040] Chaubert P., Baur Chaubert A.S., Sattler U., Forster U., Bornand V., Suter M., Welle M. (2010). Improved polymerase chain reaction-based method to detect early-stage epitheliotropic T-cell lymphoma (mycosis fungoides) in formalin-fixed, paraffin-embedded skin biopsy specimens of the dog. J. Vet. Diagn. Invest..

[bib0045] Cheng G.Y., Minden M.D., Toyonaga B., Mak T.W., McCulloch E.A. (1986). T cell receptor and immunoglobulin gene rearrangements in acute myeloblastic leukemia. J. Exp. Med..

[bib0050] Elenitoba-Johnson K.S., Bohling S.D., Mitchell R.S., Brown M.S., Robetorye R.S. (2000). PCR analysis of the immunoglobulin heavy chain gene in polyclonal processes can yield pseudoclonal bands as an artifact of low B cell number. J. Mol. Diagn..

[bib0055] Gentilini F., Calzolari C., Turba M.E., Bettini G., Famigli-Bergamini P. (2009). GeneScanning analysis of Ig/TCR gene rearrangements to detect clonality in canine lymphomas. Vet. Immunol. Immunopathol..

[bib0060] Keller S.M., Moore P.F. (2012). A novel clonality assay for the assessment of canine T cell proliferations. Vet. Immunol. Immunopathol..

[bib0065] Keller S.M., Vernau W., Moore P.F. (2016). Clonality testing in veterinary medicine: a review with diagnostic guidelines. Vet. Pathol..

[bib0070] Kyoda K., Nakamura S., Matano S., Ohtake S., Matsuda T. (1997). Prognostic significance of immunoglobulin heavy chain gene rearrangement in patients with acute myelogenous leukemia. Leukemia.

[bib0075] Lana S.E., Jackson T.L., Burnett R.C., Morley P.S., Avery A.C. (2006). Utility of polymerase chain reaction for analysis of antigen receptor rearrangement in staging and predicting prognosis in dogs with lymphoma. J. Vet. Intern. Med..

[bib0080] Langerak A.W., Groenen P.J., Bruggemann M., Beldjord K., Bellan C., Bonello L., Boone E., Carter G.I., Catherwood M., Davi F., Delfau-Larue M.H., Diss T., Evans P.A., Gameiro P., Garcia S.R., Gonzalez D., Grand D., Hakansson A., Hummel M., Liu H., Lombardia L., Macintyre E.A., Milner B.J., Montes-Moreno S., Schuuring E., Spaargaren M., Hodges E., van Dongen J.J. (2012). EuroClonality/BIOMED-2 guidelines for interpretation and reporting of Ig/TCR clonality testing in suspected lymphoproliferations. Leukemia.

[bib0085] Lindblad-Toh K., Wade C.M., Mikkelsen T.S., Karlsson E.K., Jaffe D.B., Kamal M., Clamp M., Chang J.L., Kulbokas E.J., Zody M.C., Mauceli E., Xie X., Breen M., Wayne R.K., Ostrander E.A., Ponting C.P., Galibert F., Smith D.R., DeJong P.J., Kirkness E., Alvarez P., Biagi T., Brockman W., Butler J., Chin C.W., Cook A., Cuff J., Daly M.J., DeCaprio D., Gnerre S., Grabherr M., Kellis M., Kleber M., Bardeleben C., Goodstadt L., Heger A., Hitte C., Kim L., Koepfli K.P., Parker H.G., Pollinger J.P., Searle S.M., Sutter N.B., Thomas R., Webber C., Baldwin J., Abebe A., Abouelleil A., Aftuck L., Ait-Zahra M., Aldredge T., Allen N., An P., Anderson S., Antoine C., Arachchi H., Aslam A., Ayotte L., Bachantsang P., Barry A., Bayul T., Benamara M., Berlin A., Bessette D., Blitshteyn B., Bloom T., Blye J., Boguslavskiy L., Bonnet C., Boukhgalter B., Brown A., Cahill P., Calixte N., Camarata J., Cheshatsang Y., Chu J., Citroen M., Collymore A., Cooke P., Dawoe T., Daza R., Decktor K., DeGray S., Dhargay N., Dooley K., Dooley K., Dorje P., Dorjee K., Dorris L., Duffey N., Dupes A., Egbiremolen O., Elong R., Falk J., Farina A., Faro S., Ferguson D., Ferreira P., Fisher S., FitzGerald M. (2005). Genome sequence: comparative analysis and haplotype structure of the domestic dog. Nature.

[bib0090] Luzzatto F., Pruneri G., Benini E., Manzotti M., Laszlo D., Martinelli G., Viale G. (2005). Angioimmunoblastic T-cell lymphoma with hyperplastic germinal centres and a high content of EBV-infected large B-cells carrying IgH chain gene monoclonal rearrangement. Histopathology.

[bib0095] Massari S., Bellahcene F., Vaccarelli G., Carelli G., Mineccia M., Lefranc M.P., Antonacci R., Ciccarese S. (2009). The deduced structure of the T cell receptor gamma locus in Canis lupus familiaris. Mol. Immunol..

[bib0100] O'Brien D., Moore P.F., Vernau W., Peauroi J.R., Rebhun R.B., Rodriguez C.O., Skorupski K.A. (2013). Clinical characteristics and outcome in dogs with splenic marginal zone lymphoma. J. Vet. Intern. Med..

[bib0105] Ponce F., Marchal T., Magnol J.P., Turinelli V., Ledieu D., Bonnefont C., Pastor M., Delignette M.L., Fournel-Fleury C. (2010). A morphological study of 608 cases of canine malignant lymphoma in France with a focus on comparative similarities between canine and human lymphoma morphology. Vet. Pathol..

[bib0110] Posnett D.N., Sinha R., Kabak S., Russo C. (1994). Clonal populations of T cells in normal elderly humans: the T cell equivalent to benign monoclonal gammapathy. J. Exp. Med..

[bib0115] Stadhouders R., Pas S.D., Anber J., Voermans J., Mes T.H., Schutten M. (2010). The effect of primer-template mismatches on the detection and quantification of nucleic acids using the 5' nuclease assay. J. Mol. Diagn..

[bib0120] Sze D.M. (2005). Clonality detection of expanded T-cell populations in patients with multiple myeloma. Methods Mol. Med..

[bib0125] Tamura K., Yagihara H., Isotani M., Ono K., Washizu T., Bonkobara M. (2006). Development of the polymerase chain reaction assay based on the canine genome database for detection of monoclonality in B cell lymphoma. Vet. Immunol. Immunopathol..

[bib0130] Tan B.T., Warnke R.A., Arber D.A. (2006). The frequency of B- and T-cell gene rearrangements and epstein-barr virus in T-cell lymphomas: a comparison between angioimmunoblastic T-cell lymphoma and peripheral T-cell lymphoma, unspecified with and without associated B-cell proliferations. J. Mol. Diagn..

[bib0135] Thalheim L., Williams L.E., Borst L.B., Fogle J.E., Suter S.E. (2013). Lymphoma immunophenotype of dogs determined by immunohistochemistry flow cytometry, and polymerase chain reaction for antigen receptor rearrangements. J. Vet. Intern. Med..

[bib0140] Valli V.E., Vernau W., de Lorimier L.P., Graham P.S., Moore P.F. (2006). Canine indolent nodular lymphoma. Vet. Pathol..

[bib0145] van Dongen J.J., Langerak A.W., Bruggemann M., Evans P.A., Hummel M., Lavender F.L., Delabesse E., Davi F., Schuuring E., Garcia-Sanz R., van Krieken J.H., Droese J., Gonzalez D., Bastard C., White H.E., Spaargaren M., Gonzalez M., Parreira A., Smith J.L., Morgan G.J., Kneba M., Macintyre E.A. (2003). Design and standardization of PCR primers and protocols for detection of clonal immunoglobulin and T-cell receptor gene recombinations in suspect lymphoproliferations: report of the BIOMED-2 Concerted Action BMH4-CT98-3936. Leukemia.

[bib0150] Vernau W., Moore P.F. (1999). An immunophenotypic study of canine leukemias and preliminary assessment of clonality by polymerase chain reaction. Vet. Immunol. Immunopathol..

[bib0155] Yagihara H., Tamura K., Isotani M., Ono K., Washizu T., Bonkobara M. (2007). Genomic organization of the T-cell receptor gamma gene and PCR detection of its clonal rearrangement in canine T-cell lymphoma/leukemia. Vet. Immunol. Immunopathol..

[bib0160] Zettl A., Lee S.S., Rudiger T., Starostik P., Marino M., Kirchner T., Ott M., Muller-Hermelink H.K., Ott G. (2002). Epstein-Barr virus-associated B-cell lymphoproliferative disorders in angloimmunoblastic T-cell lymphoma and peripheral T-cell lymphoma unspecified. Am. J. Clin. Pathol..

