# Supplementary material for: Optimisation and validation of a PCR for antigen receptor rearrangement (PARR) assay to detect clonality in canine lymphoid malignancies
Source: Vet Immunol Immunopathol. 2016 Dec;182:115–24. doi: 10.1016/j.vetimm.2016.10.008 (PMC5119497; doi:10.1016/j.vetimm.2016.10.008)
Supplement: Supplementary file 1 [file mmc1.docx]

Table S1: Original IgH primer sets

| Primer set | Primer number | Target | Primer name | Reference | Sequence | Product size | Usage in final protocol (see also Table 3) |
| --- | --- | --- | --- | --- | --- | --- | --- |
| A | 913 | IgH V FWR3 | CB1 (5’) | Burnett 2003 | 6-FAM-CAG CCT GAG AGC CGA GGA CAC | 85 – 139 bp | Retained as primer set 1 |
|  | 914 | IgH J1-2 | CB2 (3’) | Burnett 2003 | TGA GGA GAC GGT GAC CAG GGT |  |  |
| B | 913 | IgH V FWR3 | CB1 (5’) | Burnett 2003 | 6-FAM-CAG CCT GAG AGC CGA GGA CAC | 85 – 145 bp | Retained as primer set 3, 915 modified |
|  | 915 | IgH J3 | CB3(3’) | Burnett 2003 | TGA GGA CAC AAA GAG TGA GG |  |  |
| C | 916 | IgH V FWR3 | Tamura F (5’) | Tamura 2006 | 6-FAM-ACA CGG CCV TGT ATT ACT GT | 79 – 90 bp | Retained as primer set 2, 914 substituted for 917 |
|  | 917 | IgH J1-2 | Tamura R (3’) | Tamura 2006 | TGA GGA GAC GGT GAC C |  |  |
| D | 918 | IgH V FWR1 | 5’ FWR1(5’) | Gentilini 2009 | HEX-GAG GTG CAG CTG GTG GAG TCT | 309 – 378 bp | Retained as primer set 5 |
|  | 914 | IgH J1-2 | CB2 (3’) | Gentilini 2009 | TGA GGA GAC GGT GAC CAG GGT |  |  |
| E | 919 | IgH V FWR1 | 3’ FWR1 (5’) | Gentilini 2009 | HEX-GCC TCT GGA TTC ACC TTC AG | 262 – 315 bp | Retained as primer set 4, with 914 substituted for 920, and primer set 6, with 915 (modified) substituted for 920 |
|  | 920 | IgH J1-2 | J2 (3’) | Gentilini 2009 | AGG AGA CGG TGA CCA GGG T |  |  |

IgH primer sets initially tested. Sequences were taken from published literature as indicated. Following primer review, minor modifications were made to some primer sets for use in the standard PARR panel; see also Table 3 in the main text. Degenerate bases: V = A, C or G.

**Table S2: Original TCRγ primer sets**

| Primer set | Primer number | Target | Primer name | Reference | Sequence | Product size | Usage in final protocol (see also Table 3) |
| --- | --- | --- | --- | --- | --- | --- | --- |
| F | 921 | TCRγ V2&3&5&6&7 | TCRγ3 (5’) | Burnett 2003 | 6-FAM-TCT GGG RTG TAY TAC TGT GCT GTC TGG | 70 – 100 bp | Not used |
|  | 922 | TCRγ Jx-1 | TCRγ2 (3’) | Burnett 2003 | GTT ACT ATA AAC CTG GTA AC |  |  |
|  | 923 | TCRγ Jx-2 | TCRγ1 (3’) | Burnett 2003 | ACC CTG AGA ATT GTG CCA GG |  |  |
| G | 924 | TCRγ V2&6 | Vγa (5’) | Yagihara 2007 | 6-FAM-CGT GTA CTA CTG CGC TGC CTG G | 60 – 80 bp | Not used |
|  | 926 | TCRγ Jx-1 | Jγa (3’) | Yagihara 2007 | TAC CTT CTG YAA ATA TCT TGA |  |  |
| H | 924 | TCRγ V2&6 | Vγa (5’) | Yagihara 2007 | 6-FAM-CGT GTA CTA CTG CGC TGC CTG G | 60 – 80 bp | Not used |
|  | 927 | TCRγ Jx-2 | Jγb (3’) | Yagihara 2007 | TGT GCC AGG ACC AAG CAC TTT GTT |  |  |
| I | 925 | TCRγ V3&5&7 | Vγb (5’) | Yagihara 2007 | 6-FAM-GGC TGT ATT ACT GTG CCT GCT GG | 60 – 80 bp | Retained as primer set 10, 925 modified, 929 substituted for 926 |
|  | 926 | TCRγ Jx-1 | Jγa (3’) | Yagihara 2007 | TAC CTT CTG YAA ATA TCT TGA |  |  |
| J | 925 | TCRγ V3&5&7 | Vγb (5’) | Yagihara 2007 | 6-FAM-GGC TGT ATT ACT GTG CCT GCT GG | 60 – 80 bp | Retained as primer set 8, 925 modified, 930 (modified) substituted for 927 |
|  | 927 | TCRγ Jx-2 | Jγb (3’) | Yagihara 2007 | TGT GCC AGG ACC AAG CAC TTT GTT |  |  |
| K | 928 | TCRγ V2&3&5&6&7 | dTCRγ-Va (5’) | Chaubert 2010 | 6-FAM-GGC GTG TAC TAC TGC GCT GCC | 55 – 82 bp | Retained as primer set 9 |
|  | 929 | TCRγ Jx-1 | dTCRγ-Ja (3’) | Chaubert 2010 | TAC CTT CTG TAA ATA TCT TGA TC |  |  |
| L | 928 | TCRγ V2&3&5&6&7 | dTCRγ-Va (5’) | Chaubert 2010 | 6-FAM-GGC GTG TAC TAC TGC GCT GCC | 55 – 82 bp | Retained as primer set 7, 930 modified |
|  | 930 | TCRγ Jx-2 | dTCRγ-Jb (3’) | Chaubert 2010 | TGT GCC AGG ACC AAG CAC TTT |  |  |

TCRγ primer sets initially tested. Sequences were taken from published literature as indicated. Following primer review, minor modifications were made to some primer sets for use in the standard PARR panel; see also Table 3 in the main text. Degenerate bases: R = A or G; Y = C or T.
